# The occurrence of and factors associated with mental ill-health amongst humanitarian aid workers: A systematic review and meta-analysis

**DOI:** 10.1371/journal.pone.0292107

**Published:** 2024-05-15

**Authors:** Lily Cameron, Mary McCauley, Nynke van den Broek, Hannah McCauley

**Affiliations:** 1 Centre for Maternal and Newborn Health, Liverpool School of Tropical Medicine, Liverpool, United Kingdom; 2 Liverpool Women’s Hospital NHS Foundation Trust, Liverpool, United Kingdom; 3 Independent Consultant in Global Health, United Kingdom; University of the Witwatersrand, SOUTH AFRICA

## Abstract

**Background:**

Humanitarian crises and disasters affect millions of people worldwide. Humanitarian aid workers are civilians or professionals who respond to disasters and provide humanitarian assistance. In doing so, they face several stressors and traumatic exposures. Humanitarian aid workers also face unique challenges associated with working in unfamiliar settings.

**Objective:**

To determine the occurrence of and factors associated with mental ill-health among humanitarian aid workers.

**Search strategy:**

CINAHL plus, Cochrane library, Global Health, Medline, PubMed, Web of Science were searched from 2005–2020. Grey literature was searched on Google Scholar.

**Selection criteria:**

PRISMA guidelines were followed and after double screening, studies reporting occurrence of mental ill-health were included. Individual narratives and case studies were excluded, as were studies that reported outcomes in non-humanitarian aid workers.

**Data analysis:**

Data on occurrence of mental ill-health and associated factors were independently extracted and combined in a narrative summary. A random effects logistic regression model was used for the meta-analysis.

**Main results:**

Nine studies were included with a total of 3619 participants, reporting on five types of mental ill-health (% occurrence) including psychological distress (6.5%-52.8%); burnout (8.5%-32%); anxiety (3.8%-38.5%); depression (10.4%-39.0%) and post-traumatic stress disorder (0% to 25%). Hazardous drinking of alcohol ranged from 16.2%-50.0%. Meta-analysis reporting OR (95% CI) among humanitarian aid workers, for psychological distress was 0.45 (0.12–1.64); burnout 0.34 (0.27–0.44); anxiety 0.22 (0.10–0.51); depression 0.32 (0.18–0.57) and PTSD 0.11 (0.03–0.39). Associated factors included young age, being female and pre-existing mental ill-health.

**Conclusions:**

Mental ill-health is common among humanitarian aid workers, has a negative impact on personal well-being, and on a larger scale reduces the efficacy of humanitarian organisations with delivery of aid and retention of staff. It is imperative that mental ill-health is screened for, detected and treated in humanitarian aid workers, before, during and after their placements. It is essential to implement psychologically protective measures for individuals working in stressful and traumatic crises.

## Background

Humanitarian crises and disasters world-wide has risen dramatically in recent years [1]. In 2022, it was estimated that 274 million people needed humanitarian assistance and protection to meet human physical, psychological and social needs and to relieve suffering [2, 3]. The physical, psychological and social needs resulting from humanitarian crises often exceed available resources and overwhelm local capacity in the country affected [4]. Increasingly, affected populations rely upon the global community to offer assistance in disaster related challenges [5].

Humanitarian aid workers use their time and skills to respond to the human costs of disasters working with non-governmental organisations (NGOs) [6, 7]. Humanitarian aid workers can be local, national or international. They offer varied skills and experience, ranging from logistical support such as drivers, to highly skilled medical healthcare providers [7]. In the 2018 State of the Humanitarian System study, it was estimated that 570,000 individuals were employed by humanitarian agencies across different country settings [8]. Each humanitarian disaster is context specific and therefore the activities of a humanitarian aid worker can vary but generally encompasses the protection of civilians, as well as the delivery of food, water, healthcare and shelter, coordination and management of activities [3]. Working in hostile environments, such as in the aftermath of a natural disaster or ongoing conflict, is extremely challenging and dangerous [9]. It is well recognised that humanitarian aid workers can experience physical threats including exposure to gunfire, shelling and bombing, injury by landmines and road traffic accidents [10]; and exposure to traumatic events is a stressful occurrence. Other challenges described include physically demanding working conditions; excessive workload, chronic fatigue; lack of adequate resources and logistical support; interpersonal conflict among team members; and navigating moral and/or ethical dilemmas [11]. It is well recognised that exposure to stressors (acute and chronic) negatively affect humanitarian aid workers’ mental health and can lead to short and/or long term adverse mental health [5, 10, 12–18]. Individual studies have described that different types of humanitarian aid workers are at increased risk of developing depression [12, 13, 15, 19], anxiety [12, 13, 15, 19], burnout [12, 13, 15] and post-traumatic stress disorder (PTSD) [12–14, 19], in addition to hazardous alcohol consumption as a coping mechanism [19, 20].

In addition to the adverse effects on mental health for people working in humanitarian settings, research has demonstrated that distress in humanitarian workers can negatively impact work efficiency and efficacy and can lead to poor-decision making in the field [21]. There are also growing concerns regarding attrition rates of staff from NGOs leading to excess expenditure for organizations, as they must recruit, train and deploy new staff [8]. The main focus in humanitarian settings is often on physical safety and security but organizations are becoming more aware that there is a need to put mental health support in place and to understand what the key risk factors for mental ill-health among their staff are [1, 10].

We conducted a systematic review and meta-analysis 1) to assess the occurrence of mental ill-health among humanitarian aid workers and 2) to explore reported factors associated with mental ill-health in humanitarian aid workers.

## Material and methods

### Search strategy

CINAHL plus, Cochrane library, Global Health, Medline, PubMed and Web of Science databases were searched. Google Scholar was used to search grey literature. Broad keywords were developed and used to generate a sensitive yet specific advanced search using humanitarian aid worker and burnout, anxiety, depression and PTSD. A preliminary search showed that these mental ill health conditions were the most reported in the literature. Medical Subject Heading (MeSH) terms were added to increase the number of results and widen the scope of the search (**Table 1**). The search included studies conducted worldwide, in all languages published between 2005 and 2020. This timeline was used due to the formation of the Cluster Approach in 2005 [22]. Searches were carried out following the Preferred Reporting Items for Systematic Reviews and Meta-Analysis (PRISMA) guidelines [23]. In addition to database searching, manual reference and key author publication searching were carried out.

**Table 1:** Summary table of included studies.

Author(s) and year	Title and language	Aims/Objectives	Study design	Sending country	Destination country	Job role of humanitarian aid workers	Sample size (n = number included in study)	Screening and diagnostic tools	Psychological distress	Burnout	Anxiety	Depression	PTSD
									n = number of participants screened positive for each mental health disorder				
Armagan, E. *et al*. *2006*	Frequency of post-traumatic stress disorder among relief force workers after the tsunami in Asia: do rescuers become victims? English	To identify the frequency of PTSD among professionals who participated in the relief efforts after the 2004 Earthquake and Tsunami in Asia and to analyse the factors contributing to PTSD	Cross-sectional	Turkey	Indonesia	Doctors (45.4%), nurses (36.5%) and logistics workers (18.1%)	33	CAPS-1 interview					8 (24.4%)
Cardozo, B. L. *et al*. *2005*	The mental health of expatriate and Kosovar Albanian humanitarian aid workers English	To study the consequences of exposure to traumatic events and the risk factors for psychological morbidity among expatriate and Kosovar Albanian humanitarian aid workers	Cross-sectional	54.8% from Australia, Canada, New Zealand, Republic of Ireland, UK or US, 26.8% from other European countries	Albania	Managerial/ Administration (67.1%) and technical–health, water, logistics, other (32.9%)	605	HTQ HSCL-25 GHQ-28			71 (11.7%)	103 (17.1%)	24 (4%)
Cardozo, B. L. *et al*. *2012*	Psychological distress, depression, anxiety, and burnout among international humanitarian aid workers: a longitudinal study English	To provide scientific evidence that work and job-related stressors are associated with mental distress and burnout, and risk and mitigating factors moderate the impact of such stressors among expatriate humanitarian aid workers	Longitudinal			At outset: Head of mission/regional director (0.9%), manager/coordinator (29.3%), technical programming (34%), logistics (13.7%), administration (9.9%), other (12.3%)	Pre–deployment 212 Post deployment 169 Follow-up 154	HSCL-25 MBI-HSS	Pre = 14 (6.5%) Post = 25 (14.7%) Follow-up = 27 (17.7%)	Pre = 18 (8.53%) Post = 35 (20.71%) Follow-up = 29 (19.08%)	Pre = 12 (3.8%) Post = 20 (11.8%) Follow-up = 12 (7.8%)	Pre = 22 (10.4%) Post = 33 (19.5%) Follow-up = 31 (20.1%)	
Eriksson, C. B. *et al*. *2009*	Social support, organisational support, and religious support in relation to burnout in expatriate humanitarian aid workers English	To enrich the understanding of job-related distress and burnout in a sample of humanitarian aid workers in a faith-based agency	Cross-sectional	34 different countries of origin: Africa 23.4%, Asia 11.7%, 0.9%, Western Europe 18%, Australia/NZ 9%, North America 18.9% and Latin America 3.6%	44 different countries: African Continent 46%, Eastern Europe 20%, Asia 17%, Middle East 6%, Latin America 4%	Middle managers	111	MBI-HSS		26 (23%)			
Guimaro, M. S. *et al*. *2013*	Post-traumatic stress disorder symptoms among professionals during humanitarian aid in Haiti after the earthquake in 2010 Spanish	To assess the magnitude of the problem of PTSD in humanitarian workers and emphasise the need for evaluation and prevention of mental health consequences	Cross-sectional	Brazil, Ecuador and US	Haiti	Doctors (31.6%), nurses (37.6%), physiotherapists (9.9%), psychologists (5.3%), maintenance (3%), technical (5.1%), other (7.5%)	66	IES-R					Not detected
Jachens, L. *et al*. *2019a*	Effort-reward imbalance and burnout among humanitarian aid workers English	To identify the prevalence of burnout in a large international sample of expatriate and local humanitarian aid workers	Cross-sectional	Organization based in Geneva, 45.2% of European heritage			1980	ERI MBI-HSS PCL-6 STSS		624 (32%)			
Jachens, L. *et al*. *2019b*	Effort-Reward Imbalance and Job Strain: A Composite Indicator Approach English	To compare the ability of the two leading models—JDC-S and ERI—to identify humanitarian aid workers reporting psychological distress and to consider whether a combination of the two models might offer a superior means by which to identify workers that may benefit from targeted health protection and promotion activities	Cross-sectional				282	ERI JCQ GHQ-28	149 (52.8%)				
Musa, S. A. *et al*. *2008*	Psychological problems among aid workers operating in Darfur English	To identify psychological health problems suffered by aid workers assisting victims in Darfur, and to explore the relationship between these problems and burnout and job satisfaction rates among aid workers	Cross-sectional				53	ProQoL RWBQ GHQ-28					13 (25%)
Strohmeier, H. *et al*. *2018*	Factors associated with common mental health problems of humanitarian workers in South Sudan English	To examine symptom burden and predictors of posttraumatic stress disorder (PTSD), depression, anxiety, hazardous alcohol consumption, and burnout among humanitarian workers in South Sudan	Cross-sectional		South Sudan	Country director/head of mission (6.5%), manager/coordinator (34.7%), technical/programme (22.7%), administrative (7.6%), HR (4.3%), other (18.8%)	277	PCL-5 HSCL-25 Audit-C MBI-HSS HTQ		66 (24%)	105 (38.5%)	108 (39%)	66 (24%)

### Inclusion and exclusion criteria

All studies which reported occurrence of mental ill-health assessed in humanitarian aid workers were included. Studies reporting only historical or biographical narratives or individual case studies were excluded. Studies investigating outcomes in civilian victims of humanitarian crisis or non-humanitarian aid workers (for example military personnel) were also excluded.

### Screening, selection and data extraction

Two researchers independently screened all titles and abstracts. Evaluation of full-text studies was done independently by two researchers with reasons for exclusion recorded and any discrepancies were discussed with a third researcher. There was good agreement and similar ratings between the researchers regarding inclusion or exclusion of papers and only four papers required a third researcher review and opinion to reach consensus. Information was extracted into a pre-designed summary table and included description of the study and setting and data on the screening and/or diagnostic tools used. Additionally, data on the occurrence of the following were extracted: psychological distress, burnout, anxiety, depression and PTSD as described and reported by the authors. Throughout the reviewing and extraction processes, studies where uncertainty existed were discussed by all researchers to reach a consensus.

### Quality assessment

The Crowe Critical Appraisal Tool (CCAT) version 1.4 was used to perform quality appraisal of the studies eligible for inclusion. The CCAT was developed to overcome recognised limitations of existing critical appraisal tools (CATs) [24]. For example, most CATs appraise either one or a small number of research designs, as well as lacking the depth needed to rigorously appraise research [24]. The developers of the CCAT also recognised that a limited number of CATs have readily available validity and reliability data [24]. The CCAT was designed to be used in all research designs in health [25]. The CCAT consists of eight categories, which are further divided into 22 items and 98 item descriptors [24]. Studies are given a score for each category and scores are combined to give an overall score [24]. Each study was appraised using the CCAT Form and the CCAT User Guide together as advised by the authors, in order to ensure the most accurate scoring possible.

### Ethics approval and consent to participate

This systematic review did not involve contact with any human participants, and therefore ethical approval was not required. This study was conducted in compliance with the established ethical guidelines of the Declaration of Helsinki.

### Data synthesis

Narrative synthesis was used to summarise the findings including for associated risk factors identified and as reported in the studies. The key findings of the studies were collated into a textual narrative, and differences between the characteristics of each study were compared [26]. To conduct a meta-analysis for each of the five outcomes reported (psychological distress, burnout, anxiety, depression and PTSD) a random effects logistic regression model was applied to the data using Stata version 14.2 to estimate the underlying odds (with 95% confidence interval) of the outcome, with study as the only (random) term use in the model [27]. The estimated odds of failure and the variance of the random effects are both reported on the odds scale; additionally, the odds are reported on the natural probability scale.

## Results

**Fig 1:**
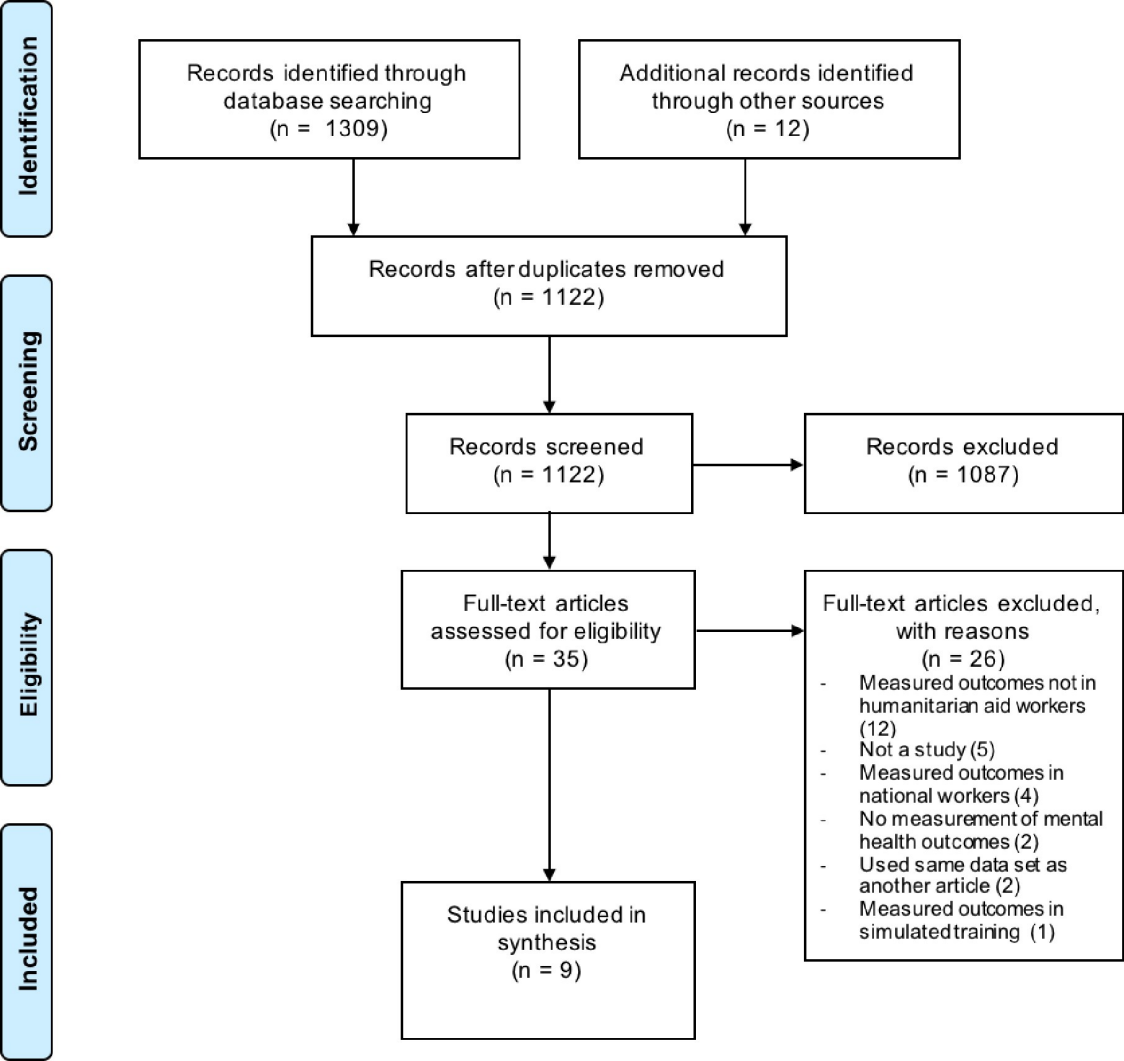
PRISMA flow diagram. Mental ill-health amongst humanitarian aid workers: Study Selection for Review.

The database search yielded 1309 results and a further 12 papers were identified through reference and author searching. After exporting all the references to EndNote X9, duplicates were removed to leave 1122 records. The remaining articles were screened, first by title and then by abstract, using application of the inclusion and exclusion criteria. Of these articles, 1087 did not meet the inclusion criteria, meaning that the majority were excluded. The full texts of the 35 articles not excluded by abstract screening were downloaded. Google Translate was used to translate two articles that were not published in English [28, 29]. The inclusion and exclusion were applied to the full-text articles. After review 26 full text papers were excluded and nine papers were established as eligible for inclusion in the review (**Fig 1 –**PRISMA flow diagram. Mental ill-health amongst humanitarian aid workers: Study Selection for Review). Of the nine papers included most papers were assessed to be of high quality with a score of 70%–93% obtained. One paper received a score of 63% as consideration of ethical matters was not reported (**Table 2**).

**Table 2:** Summary statistics for percentage (95% CI).

Outcome used	Number of studies	Total participants	Proportion (95% CI)		Odds (95% CI)		Variance (95% CI)
**Psychological distress**	2	451	30.81	(10.80–62.09)	0.45	(0.12–1.64)	0.85 (0.11–6.50)
**Burnout**	4	2537	25.61	(21.06–30.77)	0.34	(0.27–0.44)	0.04 (0.01–0.26)
**Anxiety**	3	1051	18.20	(8.88–33.71)	0.22	(0.10–0.51)	0.50 (0.09–2.69)
**Depression**	3	1051	24.07	(15.08–36.13)	0.32	(0.18–0.57)	0.24 (0.04–1.33)
**PTSD**	5	1034	9.61	(2.80–28.22)	0.11	(0.03–0.39)	1.92(0.34–10.96)

### Study population

Five studies surveyed international humanitarian aid workers only [28, 30–33] and four studies assessed both national and international aid workers [19–21, 34]. The most common term used to describe the participants was ‘humanitarian aid worker’. Other terms included: ‘relief force worker’, ‘aid worker’ and ‘humanitarian worker’. Six studies provided details of the job roles of the humanitarian aid workers who participated [19, 20, 28, 30, 31, 33]. The most featured professionals were managerial or coordination staff [19, 20, 31, 33]. Two studies included healthcare providers such as doctors, nurses, physiotherapists and psychologists [28, 30]. Other job areas included were logistics [20, 30, 31], technical [19, 20, 28, 31], administration [19, 31], maintenance [28]. A total of 3,619 humanitarian workers were assessed across the nine included studies of which 1,774 were international humanitarian aid workers. This provided a total of 6,124 assessments for inclusion in the meta-analysis (**Table 2**).

### Geographical location

Five studies included details of the country of origin of its participants [20, 21, 28, 30, 33]. Many of the studies involved individuals from several countries. High income countries were the most represented, with most of the participants originating from the United States of America (USA) and Western Europe. Five studies included information regarding the destination countries of the participants including: Indonesia [30]; Albania [20]; Haiti [28]; Sudan [34]; and South Sudan [19]. In three of these studies, the participants were working in a war or conflict setting; the conflicts in Sudan [34], South Sudan [19] and Kosovo [20]. In the other two studies, the setting was the aftermath of a natural disaster; the 2010 Haiti earthquake [28], and the 2004 Indian Ocean tsunami [30].

### Study design and data collection methods

Eight studies were cross-sectional [19–21, 28, 30, 32–34], and one study was a cohort longitudinal study. Seven studies collected data via a survey or questionnaire [19–21, 28, 31, 32, 34]. In two studies the survey was accessed via email and completed online [21, 32]. Two studies used survey interviews to collect data face-to-face [30, 33].

Different types of screening or diagnostic tools were used to assess mental health status in the included studies (**Table 3**) The Maslach Burnout Inventory-Human Services Survey (MBI-HSS) was most used to assess burnout, appearing in four studies [19, 21, 31, 33]. The MBI-HSS uses three subscales; emotional exhaustion (EE), depersonalisation (DP) and personal accomplishment (PA), to calculate burnout risk [35]. The cut-off scores for identifying high risk for burnout are: EE >27; DP >13; and PA <31 [35]. The Relief Worker Burnout Questionnaire, which uses the participant’s score to indicate the likelihood of burnout [36], was used in one study [34]. In this questionnaire, a score of 16–25 suggests suffering from work stress, 26–35 suggests possible burnout and >35 indicates probable burnout. The General Health Questionnaire (GHQ-28) and the Hopkins Symptom Checklist (HSCL-25) were used to screen for anxiety, depression and psychological distress in five studies [19, 20, 31, 32, 34]. The GHQ-28 measures emotional distress using four subscales to determine ‘psychiatric caseness’ [37, 38]. When using the binary scoring method, any score exceeding 4/28 is classified as achieving ‘psychiatric caseness’.

**Table 3:** Description of data collection tools used to assess psychological morbidity.

No.	Data collection tools to assess burnout	International abbreviation	Original country, author and date
1.	Maslach Burnout Inventory-Human Services Survey	MBI-HSS	USA, Maslach 1981 [35]
2.	Relief Worker Burnout Score		USA, Ehrenreich 2001 [36]
**No.**	**Data collection tools to assess anxiety/depression/psychological distress**	**International abbreviation**	**Original country, author and date**
1.	General Health Questionnaire	GHQ-28	UK, Goldberg 1978 [52]
2.	Hopkins Symptom Checklist	HSCL-25	USA, Parloff 1954 [53]
**No.**	**Data collection tools to assess PTSD**	**International abbreviation**	**Original country author and date**
1.	Clinician-Administered PTSD Scale	CAPS-1	USA, Blake 1990 [54]
2.	Harvard Trauma Questionnaire	HTQ	USA, Mollica 1987 [55]

The HSCL-25 is a commonly used screening tool for anxiety and depression, in which the score is used to indicate anxiety or depression caseness [39]. A score of 1.75 or more in each of the anxiety or depression subsections indicates caseness. Five studies investigated PTSD using the following tools: the Clinician-Administered PTSD Scale (CAPS-1) [30], the Harvard Trauma Questionnaire (HTQ) [20], the Impact of Event Scale (IES-R) [28], the Professional Quality of Life (ProQol) scale [34], and the PTSD Checklist for DSM (PCL-5) [19]. The Audit-C test to assess for hazardous drinking was used in one study [19]. All data collection tools have been validated in different settings [40–47] (**Table 3**).

### Occurrence of mental ill-health

Two papers reported on psychological distress [24, 25]. One longitudinal study reported 6.5% occurrence of psychological distress before deployment and up to 17.7% following deployment among a range of staff including managers, coordinators, technical and logistics staff [24, 25]. One study used two models to assess psychological distress including the Job Demand-Control-Support (JDC-S) and Effort-Reward Imbalance (ERI) models with more than half of the participants sampled (52.8%) reporting psychological distress, and one third reporting high ERI and high job strain [25]. This study reported that two models used in combination offered a superior estimation of the likelihood of psychological distress than one model alone [25]. An increased risk of burnout was described in five studies with a range of occurrence between 8.5% and 32% reported [19, 21, 31, 33, 34]. The highest occurrence was reported in a study including 1,980 international and local humanitarian aid workers of an international organization that operates in more than 100 locations worldwide [16]. Three studies reported on anxiety [19, 20, 31] which ranged from an occurrence of 3.8% pre-deployment [24] and 7.8% to 38.5% [13] during and after deployment. The same three studies also assessed depression [19, 20, 31] with an assessed occurrence of 10.4% pre-deployment [24] and 17.1% to [15] 39% during and after deployment [19, 20].

PTSD was assessed in five of the included studies [13, 15, 21, 23, 27]. Results ranged from no occurrence [21] to 25.0% [27]. One study used the Impact of Event Scale—Revised (IES-R) to screen for symptoms of PTSD with no occurrence detected amongst 66 humanitarian aid workers following an earthquake in 2010 [28]. Three studies [13, 23, 27] provided similar levels of occurrences with 1 in 4 participants screening positive for PTSD. Hazardous drinking was measured as a negative mental health outcome in two studies [19, 20]. One study reported that 46 of 284 (16.2%) of international aid workers reported drinking alcohol at hazardous levels [20] and another study reported that 50% of male and 41% of female international aid workers reporting drinking levels suggestive of disorder [19].

### Meta-analysis

Between two and five studies reported on each of the five types of mental ill-health identified (**Table 2**). Among humanitarian aid workers the odds ratio (OR) (95% CI) for psychological distress was 0.45 (0.12–1.64); burnout 0.34 (0.27–0.44); anxiety 0.22 (0.10–0.51); depression 0.32 (0.18–0.57) and PTSD 0.11 (0.03–0.39) (**Table 2**).

### Factors associated with mental ill-health

Exposure to traumatic events was reported as being associated with an increase of mental ill-health in four studies [19, 20, 30, 31] (**Table 4**). A significant association between exposure and negative mental health outcomes was found in three of the studies, with higher levels of hazardous alcohol consumption, depression and PTSD found in those with higher levels and/or frequency of trauma exposure [19, 20, 31]. Poor living conditions, security concerns, heavy workload, poor social support networks and lack of communication were identified as important ‘stressors’ and were found to impact the mental health of international humanitarian aid workers [24]. Three studies measured stress exposure, with increased risk of psychological distress, anxiety, depression and PTSD reported in humanitarian aid workers with higher chronic stress levels [19, 21, 31]. Three studies provided information regarding the pre-deployment mental health, or pre-existing mental health conditions, with a range of 9.4–19.3% of humanitarian workers self-reporting a history of mental illness [19, 20, 31]. Two found a significant association between a pre-existing psychiatric history and negative mental health outcomes post-deployment [20]. A history of mental illness was found to be positively associated with depression and non-specific psychiatric morbidity, and negatively associated with anxiety [20, 31]. Younger compared to older humanitarian aid workers were reported to be more at risk of developing mental ill-health, including depression and burnout [20, 21, 33]. Four studies investigated gender as a factor [19, 21, 30, 34], with female humanitarian aid workers more at risk of PTSD, burnout and anxiety than male humanitarian aid workers [19, 21, 30, 34]. However, male humanitarian aid workers were more likely to report hazardous alcohol consumption [19, 20]. Only three studies reported on support mechanisms and one study referenced pre-deployment training or post-deployment debrief [20, 31, 33]. International humanitarian aid workers who received high levels of support from their organisation reported lower rates of depression and burnout [20, 33] (**Table 4**).

**Table 4:** Details of factors associated with mental ill-health.

Factors associated with mental ill health	Type of associated mental ill- health
Exposure to traumatic events	Depression, PTSD, hazardous alcohol consumption [19, 20, 30, 31]
Exposure to stressors	Psychological distress, anxiety, depression, PTSD [19, 31, 32]
Pre-existing psychiatric history	Depression, anxiety [19, 20, 31]
Younger age	Depression, burnout [20, 30, 32–34]
Female gender	PTSD, burnout, anxiety [19, 30, 32, 34]
Male gender	Hazardous alcohol consumption [19, 20]
Organizational support	Negatively associated with depression, burnout [20, 31, 33]

## Discussion

### Key findings

Humanitarian aid workers experience negative mental ill-health (psychological distress, burnout, anxiety, depression and PTSD) as a result of their experiences providing relief to vulnerable populations as part of humanitarian aid work. Associated factors for mental ill-health include exposure to traumatic events, younger age, and being female. More men reported drinking alcohol at a hazardous level. Support from organisations resulted in lower rates of burnout and depression.

### Strengths and limitations

To our knowledge, this is the first systematic review and meta-analysis to focus on mental ill-health outcomes in humanitarian aid workers. The searches were not limited by geographical location or language, further increasing the chances for all relevant literature to be identified. However, it was not possible to approach researchers directly who were working in regions affected by major conflict and human displacement for any unpublished data. The search was conducted for paper published from 2005 and 2020. This timeline was used because in 2005 the Emergency Relief Coordinator and the Inter-Agency Steering Committee (IASC) initiated the reform of the humanitarian system, resulting in the formation of the Cluster Approach [22]. Although all the screening tools used are validated, the variety of screening tools used across the included studies must be considered when interpreting the results of this review. We acknowledge that this is a limitation and highlight the need for standardisation of assessment tools and international agreement on cut-off points while appreciating that this is the real-life pragmatic approach currently taken by organisations as well as researchers. Furthermore, all but two of the data collection tools were self-reported questionnaires, with no clinical follow-up to confirm diagnoses.

### Interpretation of findings

Although many of the included studies followed the same trends, there was variation in the rates of mental ill-health reported. In addition to variation in screening tools as well as timing of screening, a further explanation for this could be the country of origin of the humanitarian aid workers. Cultural differences have been shown to have implications for mental ill-health, including how mental illness is viewed, if and how it is reported and how treatment is accessed [48]. Although not all the included studies gave information regarding the nationalities of their participants, most of the humanitarian aid workers sampled were from high-income countries. One explanation for the high rates of burnout in international workers maybe that international aid workers work in an unfamiliar setting. This paired with the unrealistic expectations of humanitarian aid work, can lead to emotional exhaustion and burnout [21]. National workers, by comparison, can often relate to the victims of disaster and may even have been affected by humanitarian crisis themselves. Therefore, providing humanitarian aid could lead to further trauma for national workers, which could explain why they are more at risk of PTSD and other mental ill-health outcomes such as anxiety [7]. Different settings of deployment, such as natural disasters or conflict zones, present different challenges for the delivery of aid, which could lead to different mental health impacts for affected humanitarian aid workers. It has been suggested that people tend to be more frustrated about disasters that are caused by humans, such as armed conflict [49]. Some research has shown that the psychological impacts of man-made disasters can be more pronounced than those of natural disasters such as earthquakes or floods [50].

Pre-existing mental ill-health have been shown to be a risk factor for the development of new psychological morbidity following disasters [51]. Therefore, it was surprising that only three of the included studies collected data on previous mental ill-health of their participants. An explanation for the positive association between younger age and increased negative mental ill-health could be that older humanitarian aid workers have developed more effective coping strategies through their life experiences [21]. However, it could be that the older individuals sampled are those that have chosen to stay in the humanitarian sector [33]. Others may have in fact experienced negative mental ill-health, and not returned to humanitarian work as a result. Findings regarding the significance of gender as a risk factor were inconsistent in this review. Research has shown that gender has a role to play for mental ill-health, so although overall rates of psychiatric disorders are almost the same in men and women, there are big differences in the patterns of mental illness between the genders [52]. For example, female humanitarian aid workers can be more exposed to risk factors for negative mental ill-health, such as sexual harassment and gender-based violence [19].

The benefits of adequate training for humanitarian aid workers are well documented. Pre-deployment training can prepare individuals for the challenges they may face while working in a disaster setting and can help to build resilience [53]. Team building exercises have also been shown to be useful in strengthening communication, which can be protective against organisational stressors [18].

The literature regarding post-deployment debriefing was more inconclusive. Debriefing is thought to reduce the impact of trauma by allowing an individual to reconstruct their experiences [5], and it has been shown to reduce distress and anxiety [17]. However, there is some evidence that debriefing is in fact unhelpful, and that focusing on the traumatic incident should not be routine practice [45]. With this evidence in mind, both the Inter-Agency Standing Committee (IASC) and the Antares Foundation have developed guidelines outlining key actions for organisations to address the psychological needs of the humanitarian aid workers they employ [54, 55].

### Implications of findings

Mental ill-health has a significant negative impact on an individual’s personal well-being, and on a larger scale can reduce the efficacy of humanitarian organisations with delivery of aid and retainment of staff. A pre-existing mental health condition is a risk factor for the development of further psychological problems in humanitarian aid workers, and it is essential that appropriate screening is conducted prior to deployment. Individuals who are more at risk should be provided with extra support and follow up management. Education should be provided to all prospective humanitarian aid workers regarding the potential negative psychological impacts of working in a humanitarian setting. Pre-deployment training can greatly improve the resilience and overall preparation of humanitarian aid workers [5, 17, 18, 53] and it is important that humanitarian organisations follow all available guidelines and provide adequate pre-deployment training for of all their employees. Psychological support systems need to be in place for humanitarian aid workers both during and after deployment. Care needs to be taken to reduce the workload of individual workers where possible, in order to reduce stress and subsequent stress-related mental illness. Although the evidence surrounding formal debriefing is inconclusive, support in the form of confidential counselling should be readily available to humanitarian workers at all points following deployment.

## Conclusion

Humanitarian aid workers are exposed to a range of stressors and traumatic events whilst providing aid in humanitarian emergencies, and this can result in the exacerbation of pre-existing mental ill-health and/or the development of new mental ill-health including psychological distress, burnout, anxiety, depression, and PSTD. It is imperative that mental ill-health is screened for, detected and treated for humanitarian aid workers, before, during and after their deployment. Humanitarian organisations must be responsible to prevent and manage mental ill-health by providing support (for example freely available, confidential counselling) prior to, during and post-placement. The use and need for humanitarian assistance are ever increasing and it is important that humanitarian aid workers are healthy and able to function to the highest standard. Although there appears to have been an increase in attention on the mental health of humanitarian aid workers in recent years, there is still a need to conduct further large-scale research to investigate the associations between humanitarian work and mental ill-health. Further research needs to investigate the most effective training and support systems that need to be in place for humanitarian aid workers.

## Supporting information

S1 Checklist PRISMA 2020 checklist. (DOCX)

## List of abbreviations

ALNAP: Active Learning Network for Accountability and Performance
CAPS-1: Clinician Administered PTSD Scale
CCAT: Crowe Critical Appraisal Tool
EE: Emotional Exhaustion
DP: Depersonalisation
ERI: Effort-Reward Imbalance
GHQ-28: General Health Questionnaire
HSCL-25: Hopkins Symptom Checklist
HTQ: Harvard Trauma Questionnaire
IASC: Inter-Agency Standing Committee
IES-R: Impact of Event Scale
JCQ: Job Content Questionnaire
LMIC: Low- and middle-income country
MeSH: Medical Subject Heading
MBI-HSS: Maslach Burnout Inventory-Human Services Survey
NGO: Non-governmental organization
PA: Personal Achievement
PRISMA: Preferred Reporting Items for Systematic Reviews and Meta-Analysis
ProQuol: Professional Quality of Life
PTSD: Post-Traumatic Stress Disorder
PCL-5: PTSD Checklist
RWBQ: Relief Worker Burnout Questionnaire
STSS: Secondary Traumatic Stress Scale
UNOCHA: United Nations Office for the Coordination of Humanitarian Affairs
